# Predicting brain health in older adults through automated language measures

**DOI:** 10.1002/alz70857_102831

**Published:** 2025-12-25

**Authors:** Gonzalo Nicolás Pérez, Ivan Caro, Joaquín Valdez Bisé, Franco Javier Ferrante, Lara Gauder, Alejandro Sosa Welford, Joaquín Ponferrada, Luciana Ferrer, Agustin Ibanez, Andrea Slachevsky, Adolfo M Garcia

**Affiliations:** ^1^ Consejo Nacional de Investigaciones Científicas y Técnicas (CONICET), Buenos Aires, Argentina; ^2^ Universidad de Buenos Aires, Buenos Aires, Argentina; ^3^ Cognitive Neuroscience Center, University of San Andrés, Victoria, Buenos Aires, Argentina; ^4^ Department of Psychiatry, School of Medicine, Pontificia Universidad Católica de Chile, Santiago, Chile; ^5^ University of Buenos Aires, Ciudad Autonoma de Buenos Aires, CABA, Argentina; ^6^ Institute of Computer Science, University of Buenos Aires, Buenos Aires, Argentina; ^7^ Latin American Brain Health Institute (BrainLat), Universidad Adolfo Ibáñez, Santiago, Región Metropolitana, Chile; ^8^ Cognitive Neuroscience Center (CNC), Universidad de San Andrés, Buenos Aires, Buenos Aires, Argentina; ^9^ Adolfo Ibáñez, Santiago, Chile Cognitive Neuroscience Center, Universidad de San Andrés, Buenos Aires, Buenos Aires, Argentina; ^10^ Global Brain Health Institute, Dublín, Dublín, Ireland; ^11^ Neurology Service, Department of Medicine, Clínica Alemana, Universidad del Desarrollo, Santiago, Región Metropolitana de Santiago, Chile; ^12^ Memory and Neuropsychiatric Center (CMYN) Neurology Department, Hospital del Salvador and Faculty of Medicine, University of Chile, Santiago, Region Metropolitana, Chile; ^13^ Neuropsychology and Clinical Neuroscience Laboratory (LANNEC), Physiopathology Department ‐ ICBM, Neuroscience and East Neuroscience Departments, Faculty of Medicine, Universidad de Chile, Santiago, Chile; ^14^ Geroscience Center for Brain Health and Metabolism (GERO), Santiago, Región Metropolitana de Santiago, Chile; ^15^ Global Brain Health Institute (GBHI), University of California San Francisco (UCSF); & Trinity College Dublin, Dublin, Ireland; ^16^ Faculty of Education, National University of Cuyo, Mendoza, Argentina; ^17^ Universidad de Santiago de Chile, Santiago, Santiago, Chile; ^18^ BrainLat Institute, Santiago, Santiago, Chile

## Abstract

**Background:**

With increasing life expectancy, aging‐related neurocognitive challenges are becoming more prevalent worldwide. A continuum of severity, from subjective cognitive decline (SCD) to mild cognitive impairment (MCI) and Alzheimer's disease dementia (ADD), is measurable through cognitive and neuroimaging assessments. However, these approaches are costly, subject to scheduling delays, and reliant on specialized personnel or resources, which are scarce in many regions. Automated speech and language analysis (ASLA) offers an affordable, scalable solution for detecting neurocognitive compromise in underserved areas. Yet, no prior study has employed ASLA to predict neuropsychological and brain measures across this continuum in Spanish‐speaking Latinos. Our study addresses this gap.

**Method:**

We recruited 150 Chilean individuals with diverse cognitive profiles: 17 healthy controls, 55 with SCD, 57 with MCI, and 21 with ADD. Participants completed 1‐minute phonemic and semantic fluency tasks, alongside cognitive (Addenbrooke's Cognitive Examination‐III [ACE‐III], Montreal Cognitive Assessment [MoCA]) and executive function (INECO Frontal Screening [IFS]) tests and MRI scans. Machine learning models were trained with word‐property and speech‐timing features from fluency responses (both separate and combined), derived via the TELL app, to predict cognitive test outcomes, total gray matter (GM) and white matter volumes, hippocampal GM volume, and a mask encompassing ADD‐sensitive regions. The best regressors were selected based on the 95% confidence intervals of *R* ^2^ scores. Pearson's partial correlations between actual and predicted values were computed, controlling for age, sex, and education (and MoCA scores for brain‐related measures). Analyses were repeated for each fluency task, their combination, and their average.

**Results:**

Significant partial Pearson correlations were obtained for ACE‐III (*r* = .55, *p* < .001), MoCA (*r* = .39, *p* < .001), and IFS (*r* = .31, *p* = .001) scores, as well as for normalized GM volume (*r* = .34, *p* = .006). All results correspond to word‐property features, with combined fluency tasks.

**Conclusion:**

A fully automated, multivariate pipeline captures cognitive and MRI measures of brain health based on brief fluency tasks. Unlike gold‐standard tests, this approach is examiner‐independent, objective, time‐efficient, and affordable. This approach represents a scalable resource to favor equity in the global fight against dementia and cognitive decline.

